# Automatic rehabilitation assessment method of upper limb motor function based on posture and distribution force

**DOI:** 10.3389/fnins.2024.1362495

**Published:** 2024-02-19

**Authors:** Jing Bai, Guocheng Li, Xuanming Lu, Xiulan Wen

**Affiliations:** ^1^Industrial Technology Research Institute of Intelligent Equipment, Nanjing Institute of Technology, Nanjing, China; ^2^Jiangsu Provincial Engineering Laboratory of Intelligent Manufacturing Equipment, Nanjing, China; ^3^Automation Department, Nanjing Institute of Technology, Nanjing, China

**Keywords:** rehabilitation assessment, upper limb, posture, distributed force, fuzzy logic

## Abstract

The clinical rehabilitation assessment methods for hemiplegic upper limb motor function are often subjective, time-consuming, and non-uniform. This study proposes an automatic rehabilitation assessment method for upper limb motor function based on posture and distributed force measurements. Azure Kinect combined with MediaPipe was used to detect upper limb and hand movements, and the array distributed flexible thin film pressure sensor was employed to measure the distributed force of hand. This allowed for the automated measurement of 30 items within the Fugl-Meyer scale. Feature information was extracted separately from the affected and healthy sides, the feature ratios or deviation were then fed into a single/multiple fuzzy logic assessment model to determine the assessment score of each item. Finally, the total score of the hemiplegic upper limb motor function assessment was derived. Experiments were performed to evaluate the motor function of the subjects’ upper extremities. Bland-Altman plots of physician and system scores showed good agreement. The results of the automated assessment system were highly correlated with the clinical Fugl-Meyer total score (*r* = 0.99, *p* < 0.001). The experimental results state that this system can automatically assess the motor function of the affected upper limb by measuring the posture and force distribution.

## 1 Introduction

With the acceleration of social aging, the incidence of stroke is gradually increasing. The death and disability rate of stroke is extremely high, and 70% of the survivors have varying degrees of disability (Garro et al., 2021). Motor disorders of the limbs significantly reduce patients’ quality of life and cause considerable suffering. The plasticity of the nervous system has been demonstrated by research (Turon et al., 2018). Early intervention in the early stages of the disease has the potential to reduce the severity of disability and significantly improve the patient’s quality of life (Meng et al., 2023). Rehabilitation assessment is based on the level of function, degree of damage, and recovery of stroke patients. It provides a scientific basis for formulating rehabilitation treatment programs, evaluating patients’ functional changes, and judging treatment effects and prognosis.

However, the commonly used clinical methods to assess upper limb motor function after stroke are mainly qualitative or semi-quantitative, including active mobility rating (AMR), ARAT-Brunnstrom, Fugl–Meyer rating (FMA), Barthel index, Wolf motor function test (WMFT), and so on (Wolf et al., 1989; Sanford et al., 1993; Sardari et al., 2023). The degree of limb impairment is mainly evaluated subjectively through manual measurement of angle and force information by physicians using protractors and dynamometers. The traditional scale-based assessment has been widely accepted in the medical field (Du et al., 2022), but there are still some shortcomings:

(1) The assessment mechanism is subjective, leading to variation in results. Longitudinal results for the same patient cannot be compared, let alone between different patients. (2) There is no established uniform assessment system among hospitals. (3) The process is also time-consuming. Research on quantitative automatic rehabilitation assessment methods based on automated information technology can improve the standardization of rehabilitation medical technology, reduce assessment and testing time, and alleviate the burden on rehabilitation physicians. Research into automated information technology-based rehabilitation assessment methods could improve the standardization of rehabilitation medical technology, reduce assessment testing time and relieve the burden on rehabilitation professionals.

Modern devices for automatic assessment of upper limb motion function mainly include wearable sensors, rehabilitation robots and visual motion capture systems (Ona Simbana et al., 2019). Wearable sensors mainly include inertial measurement Unit (IMU), surface electromyography (sEMG) sensor, data glove, etc. Oubre et al. (2020) used two wearable inertial sensors on the wrist and the sternum to estimate upper-limb impairment, and proposed an unsupervised clustering algorithm and a supervised regression model to estimate FMA scores. Ueyama et al. (2023) automated the FMA with 9-axis motion sensors and measured 23 FMA upper-limb items. Pan et al. (2021) proposed an evaluation method for upper limb motor function in stroke patients with five features by using the inertial sensor and sEMG sensor. Li C. et al. (2022) used data glove and Thalmic Myo armband to assess the hand motor function quantitatively. Dutta et al. (2022) developed a data glove housing 6 flex sensors, 3 force sensors, and a motion processing unit to evaluate the grasp ability of stroke patients. There is also related research on robot rehabilitation assessment. Moon et al. (2023) proposed a method for evaluating upper limb motor performance with robot based on a normal reaching movement model. However, patients need to wear the IMU and attach the sEMG sensor to their skin. Wearable IMUs are prone to displacement, and stroke may cause hand contractures in some patients, making data gloves difficult to wear. Additionally, sEMG signals are weak, random, and susceptible to interference from muscle status, skin sweat, and the environment. Robots are expensive and beyond the reach of the average family.

In terms of visual motion capture systems, VICON (Oxford, United Kingdom) is an optical motion capture system, that has become the gold standard for motion analysis (Van Crombrugge et al., 2022), but it is relatively expensive. Infrared imaging devices are also used to assess hand function (Fang et al., 2019). Kinect can visually capture three-dimensional motion and has been used in many motion analysis studies due to its comfort, low cost, easy installation, and suitability for home or community hospitals.

Bai and Song (2019) used Kinect V1 and IMU sensors to evaluate 15 FMA items, and automatically evaluated the upper limb combined with the reachable workspace of each subject. Motion measurement sensors, such as inertial and visual sensors (Ambros-Antemate et al., 2022; Francisco-Martínez et al., 2022), are unable to assess changes in stiffness. To quantify joint stiffness, force measurement is a more appropriate method. Lee et al. (2016, 2018) used Kinect v2 and force sensing resistor sensors, and developed a rule-based binary logic classification algorithm, to realize an automated FMA system for upper extremity motor function assessment. Li Y. et al. (2022) proposed an automated evaluation system composed with RealSense D435, Leap Motion and Force Sensitive Resistors.

Bai proposed less automatic motion protocol (only 15 items). Lee uses Kinect v2 sensor, resulting in inaccurate and unstable hand posture measurement, and inaccurate forearm pronation/pronation tracking. The scheme proposed by Yue Li uses three different types of sensors, and the detection system is complex. The accuracy of RealSense D435 human posture measurement can be affected by the position of the hand attitude measurement sensor and its affiliated platform. Furthermore, these studies utilized a single force sensing resistor sensor to measure hand grasping ability, which only measures thumb and index finger force and may not accurately reflect force at other hand positions.

Regarding evaluation methods, the researchers suggest utilizing machine learning techniques for data processing (Chung et al., 2022). Eichler et al. (2018) developed a multi-camera tracking system with SVM and Random Forest to evaluate the motion of stroke patients. Kim et al. (2020) proposed a method to judge the severity of elbow spasm. Machine learning algorithm was used to analyze the acceleration and rotation attributes of the affected elbow joint, and the degree of spasm was classified. Miao et al. (2021) proposed to adopt smartphone and Kinect sensor to collect upper limb movement data and use the long short-term memory neural network to evaluate upper limb movement function. Deb et al. (2022) adapt spatio-temporal GCN for the assessment of rehabilitation exercises. However, implementing machine learning, particularly deep learning, in clinical practice is challenging due to the need for extensive labeled data.

To address the issues with the previous studies, this study proposes a new approach that utilizes the array distributed flexible thin film pressure sensor (DFPS) and Azure Kinect for Upper Extremity FMA (FMA-UE) automation. Azure Kinect combined with MediaPipe can improve the accuracy of upper limb and hand posture recognition. Additionally, the DFPS is used to refine hand joint stiffness measurements. The fuzzy logic-based assessment method is adopted to avoid the problem of reliance on large amounts of labeled data.

**Figure 1:**
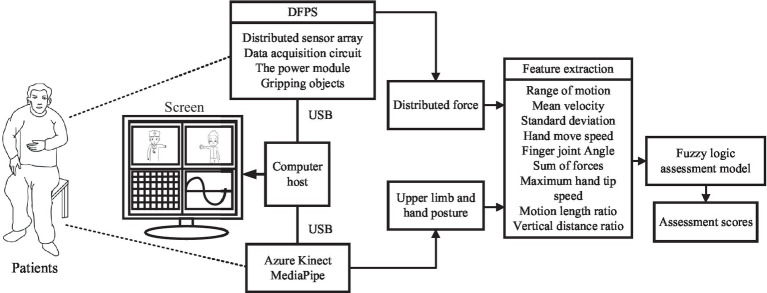
Automatic rehabilitation assessment system of upper limb motor function based on posture and distribution force.

The block diagram of the automatic rehabilitation assessment system of upper limb motor function based on posture and distributed force measurement is shown in Figure 1. The assessment protocol is presented to the subjects through a display screen. The DFPS and Azure Kinect are connected to the computer via USB. Azure Kinect combined with MediaPipe automatically recognizes the subject’s upper limb and hand joint positions, which are used to calculate information like motion angles under each assessed movement within the FMA scale. DFPS is used to measure force information in the hand during different gripping modes. Next, feature information is extracted for both the affected and healthy upper limbs and hands. The ratio or deviation is then calculated and input into a single/multiple fuzzy logic assessment model to derive assessment scores for each item in automated FMA-UE scale. Finally, the total assessment scores for the upper limbs are calculated.

## 2 Methods

### 2.1 Automatic FMA-UE

The Fugl–Meyer assessment is a widely used method for clinically evaluating post-stroke motor dysfunction. It consists of 33 items for assessing upper extremity motor function, including 18 for shoulder-arm motor function, 12 for wrist-hand motor function, and 3 for coordination. The scoring criteria for the Fugl–Meyer upper extremity motor function assessment are: the assessment is scored out of 66 points, with 2 points awarded for complete completion of each item, 1 point for partial completion, and 0 points for failure to complete. The FMA-UE score is used to classify the severity of hemiplegia, with scores below 32 indicating severe hemiplegia, scores between 32 and 57 indicating moderate hemiplegia, and scores between 58 and 66 indicating mild hemiplegia (Gladstone et al., 2002).

The FMA-UE is a detailed scale closely related to the functions required for daily living activities. It can visually and quickly reflect abnormal movement patterns, making it a comprehensive assessment. However, the clinical test is lengthy, tedious, and subjective. To effectively assess the motor function of the upper extremity in post-stroke patients, automating as many items as possible in the FMA-UE program is necessary. This study improved the testing method of the scale to enable automated measurement of upper limb motor function in stroke patients.

The method proposed in this paper contains 30 items (F1–F30) for automation, as shown in the “FMA-UE Item” column in the Table 1. Only the two items shown in grey are not included, namely the “Reflex activity” and the “Normal reflex activity,” as they require a small hammer to tap the muscles. All automatic evaluations are summarized into 21 sets of actions as shown in the “Motion” column. The actions M16–M20 (M16 is hook grasp., M17 is lateral pinch, M18 is Pincer grasp., M19 is cylinder grasp., M20 is Sphere grasp) adopt the DFPS, and other actions are measured using Azure Kinect combined with MediaPipe (AKM). The column “Feature” in the table indicates the feature information extracted for each item.

**Table 1:** Target FMA-UE items.

Category		Motion	FMA-UE item	Sensors	Feature symbol	Feature
Shoulder/elbow	Reflex activity		(1) Flexors data	N/A		
			(2) Extensors	N/A		
	Flexor synergy	M1	(3) Shoulder elevation	AKM	F1	ROM, MV, SD
			(4) Shoulder retraction	AKM	F2	
			(5) Shoulder abduction (≥90°)	AKM	F3	
			(6) Shoulder external rotation	AKM	F4	
			(7) Elbow flexion	AKM	F5	
			(8) Forearm supination	AKM	F6	
	Extensor synergy	M2	(9) Shoulder adduction/internal rotation	AKM	F7	
			(10) Elbow extension	AKM	F8	
			(11) Forearm pronation	AKM	F9	
	Volitional movement mixing synergies	M3	(12) Hand to lumbar spine	AKM	F10	Hand-to-hip motion length ratio
		M4 + M5	(13) Shoulder flexion 0–90° with the elbow fully extended	AKM	F11	ROM, MV, SD of M4 and M5
		M6 + M7 + M8	(14) Forearm pronation/supination with the elbow 90°and shoulder 0°	AKM	F12	ROM, MV, SD of M6–M8
	Volitional movement with little or no synergy	M9 + M5 + M6	(15) Shoulder abduction 0–90° with elbow fully extended and forearm pronation	AKM	F13	ROM, MV, SD of M5, M8, and M9
		M10 + M5	(16) Shoulder flexion 90–180° with elbow fully extended	AKM	F14	ROM, MV, SD of M10, and M5
		M5 + M6+ M11	(17) Forearm pronation/supination with the elbow fully extended and shoulder 30°–90°	AKM	F15	ROM, MV, SD of M5, M8, and M11
	Normal reflex activity		(18) Biceps, triceps, finger flexors	N/A		
Wrist/hand	Wrist stability	M12 + M7 + M8	(19) Wrist stability at 15° dorsiflexion with elbow 90°and shoulder 0°	AKM	F16	ROM, MV, SD of M12, M7, and M8
		M13 + M7	(20) Repeated wrist flexion and extension (WFE) with elbow 90°	AKM	F17	ROM, MV, SD of M13 and M7
		M12 + M5 + M11	(21) Wrist stability at 15° dorsiflexion with elbow 0° and shoulder 30°	AKM	F18	ROM, MV, SD of M12, M5, and M11
		M12 + M5 + M11	(22) Repeated wrist flexion and extension with elbow 0° and shoulder 30°	AKM	F19	ROM, MV, SD of M12, M5 and M11
		M13 + M5 + M11	(23) Circumduction with elbow 0° and shoulder 30°	AKM	F20	ROM, MV, SD of M5 and M11
	Hand	M14	(24) Mass flexion	AKM	F21	Max angle and MV
		M15	(25) Mass extension	AKM	F22	
		M16	(26) Hook grasp	DFPS	F23	Total force per sensing unit
		M17	(27) Lateral pinch	DFPS	F24	
		M18	(28) Pincer grasp	DFPS	F25	
		M19	(29) Cylinder grasp	DFPS	F26	
		M20	(30) Sphere grasp	DFPS	F27	
Coordination/speed		M21	(31) Finger-nose tremor	AKM	F28	The MV and MA of the fingertips
			(32) Finger-nose dysmetria	AKM	F29	Vertical distance ratio, motion length ratio
			(33) Finger-nose speed	AKM	F30	V and t

N/A, not applicable. The gray part is not included in this automated FMA-UE. Motion represents all movements; there are 20 motion tasks in total. F1–F20 represent the feature symbol. FMA-UE item represents the item included in this automated FMA-UE. AK means Azure Kinect, DFPS means the Array Distributed Flexible Thin Film Pressure Sensor. ROM means Range of motion, MV represents mean velocity, SD means the standard deviation.

### 2.2 System design

In this study, an automated FMA-UE upper limb motor function assessment system is proposed, including an Azure Kinect and a set of DFPS. The RGBD camera combined with MediaPipe enables motion and posture tracking of the upper limbs and hands. Azure Kinect Depth Camera integrates a depth sensor, a spatial microphone array, a video camera, and a direction sensor to achieve depth recognition based on the TOF principle, which can realize three-dimensional tracking of the human body and identify the position information of 32 joints (Wei et al., 2022). MediaPipe is an open-source machine learning application development framework developed by Google. MediaPipeHands is a high-fidelity hand and finger tracking solution. It uses machine learning to infer the 3D coordinates of 21 joints of a hand from a single frame.

MediaPipe uses color image to recognize human motion information. Azure Kinect’s collection of human joint points includes depth image information. Therefore, the color image and depth image need to be aligned initially. The camera can capture three types of data simultaneously, namely RGB images (three-channel images), depth images (single-channel grayscale maps), and color 3D point clouds. The device calibration data is retrieved before the coordinate system conversion can be performed. Then the 3D points of the source coordinate system are converted into the 3D points of the target coordinate system using the external calibration of the camera, and the corresponding 2D pixel coordinates are calculated using the target camera’s internal calibration to align the depth image and color image for subsequent hand tracking and upper limb joint tracking.

The DFPS comprises a sensing element, an array scanning module, a signal acquisition and processing module, and a power supply module. A piezoresistive tactile sensor is used as the sensing element, based on the semiconductor piezoresistive effect. The principle of piezoresistive tactile sensors is that the electrical resistivity of the elastomer material varies with the magnitude of the pressure, which converts the pressure signals on the contact surfaces into electrical signals. The array scan module performs periodic scans of the sensor array and stabilizes the power supply and battery voltage. The TPS62046 is used in the voltage regulator part to convert the 5 V DC power supply to 3.3 V. The scanning circuit utilizes the CD4052 analog multi-switch to cyclically supply power to the sensing unit, access the voltage divider circuit for voltage division, and transmit the resistive voltage division value of the sensing unit to the signal acquisition and processing module. The STM32 microcontroller module acquires the voltage value of the sensing unit for data interpretation and outputs the measurement results to the computer through USB to serial port. The sensor is then calibrated using the calibration device to establish the relationship between voltage and pressure.

### 2.3 Data processing

Prior to feature extraction, the raw data of the measured postural signals were filtered by a fourth-order Butterworth low-pass filter with a cut-off frequency of 12 Hz to remove artefacts caused by the patient’s voluntary movement and gravity during the measurement (Ren et al., 2020).

In order to enable doctors to quickly use the automatic evaluation method proposed in this study, the description of limb movements adopts the method in rehabilitation medicine, based on the standards defined by the International Society of Biomechanics (ISB) (Wu et al., 2005). The Standardization and Terminology Committee (STC) of the ISB proposes a definition of a joint coordinate system (JCS) for each joint (Da Gama et al., 2016). In this paper, only the coordinates of the right joints are presented, and the left joint is the mirror image of the right (with respect to the sagittal plane *z* = −*z*).

The coordinate system for the thorax is defined as follows: the *Y*_T_ axis is a unit vector from the spine chest (SC) to the neck, the ISB conventionally uses the *X*-axis turned away from the body and pointing directly anterior to the body, and the *Z*_T_ axis is a unit vector perpendicular to the *X*_T_ and *Y*_T_ axes, which can be computed by the cross product between them. The coordinate system for the right shoulder joint (CS) is also defined. For that purpose, *Y*_S_ is used as the unit vector from ER towards SR, and *Z*_S_ as the vector perpendicular to the plane formed by *X*_S_ and *Y*_S_. The flexion/extension, adduction/abduction and internal/external rotation angles of the shoulder joint can be solved using the Euler angles of the rotation matrices of the two coordinate systems. The angles of flexion and extension of the wrist joint and the anterior and posterior rotation of the forearm can also be solved. The elbow flexion angle is defined as the angle between the upper arm and the forearm, which can be determined using the flexion angle vector. F1 and F2 can be calculated using the shoulder-neck (SN) vector with respect to the horizontal and frontal planes, respectively.

The range of motion (ROM), mean velocity (MV), and standard deviation (SD) are selected as the eigenvalues for item (3–11). ROM is defined as the difference between the maximum and minimum angle of motion, MV represents the average velocity, and SD is the standard deviation of the unaffected side and the affected side. To ensure consistency across individuals, the healthy side data is used as the standard. The standard deviation (*δ*) between the unaffected and healthy side for the same motion is calculated using Eq. 1.


(1) $$\delta =\sqrt{\frac{\sum_{i=1}^{n}\Delta d_{i}^{2}}{n}}$$

where *n* is the sampling frequency of each action, and Δ*d* is the deviation between the unaffected and healthy side. The target symbol F1-F9 can extract the ROM, MV and SD of each action as feature information.

Item (12) extracts the hand-to-hip motion length ratio α (Eq. 2) as characteristic values F10.


(2) $$\alpha =\min\frac{d_{AHC}}{d_{OHC}}$$

where *d*_OHC_ is the distance from the original position of the hand to the center of the hip, *d*_AHC_ is the distance from the actual position of the hand to the center of the hip.

As shown in Table 1, item (13), (16), and (20) each contain two sub-motions, the ROM and MV and SD of each sub-motions should be extracted as F11, F14 and F17 separately. Item (14), (15), (17), (19), and (21)–(23) each contain three sub-motions. The ROM and MV and SD of each sub-motions (F12, F13, F15, F16, and F18–F20) need to be extracted separately.

Aligning the color image and depth image using Azure Kinect calibration function. Then, aligning the wrist joint position and fingertip position recognized by MediaPipe and Azure Kinect, respectively. The coordinates of other hand joints are transformed simultaneously. This allows for tracking of hand joint positions and capturing of hand posture.

**Figure 2:**
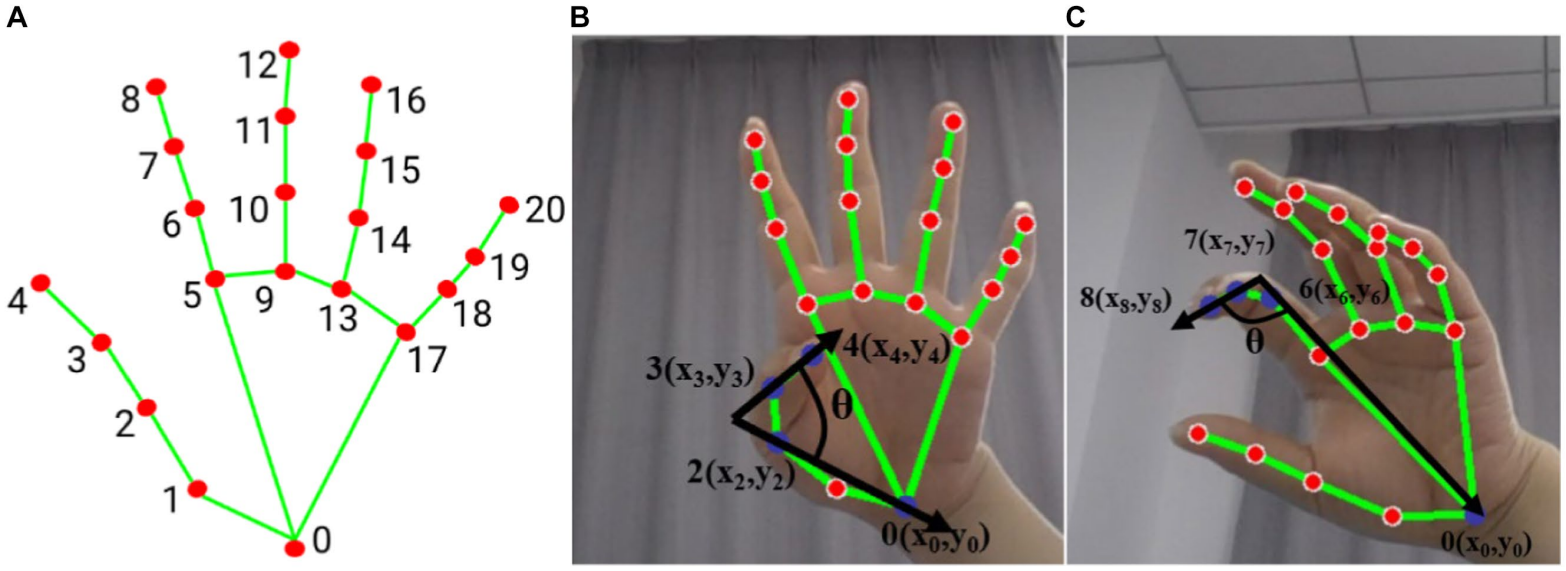
Hand posture **(A)** hand node serial number, **(B)** thumb flexion vector angle (blue dot marked as used joint), **(C)** index finger flexion vector angle (blue dot marked as used joint).

The bending angle of the finger joints was calculated by determining the maximum value of the finger’s bending angle and the MV to judge the Mass flexion (F21) and Mass extension (F22). Figure 2 shows the calculation of the bending angle of the finger joints. Panel (A) displays the ordinal number of the 21 key points of the hand, while panels (B) and (C) show the schematic of the thumb and index finger bending vector pinch angles, respectively. The thumb fingertip joint point 4 and joint point 3 form vector 3_4. The joint point 2 and wrist origin point (joint point 0) form vector 0_2. The angle between vector 3_4 and vector 0_2 is calculated according to Eq. 3.


(3) $$\theta =\arccos\left(\frac{\left(x_{4}-x_{3}\right)\left(x_{0}-x_{2}\right)+\left(y_{4}-y_{3}\right)\left(y_{4}-y_{2}\right)}{\sqrt{{\left(x_{4}-x_{3}\right)}^{2}+{\left(y_{4}-y_{3}\right)}^{2}}\times \sqrt{{\left(x_{0}-x_{2}\right)}^{2}+{\left(y_{0}-y_{2}\right)}^{2}}}\right)$$

The angle between vector 0_6, consisting of joints 0 and 6, and vector 7_8, consisting of joints 7 and 8, represents the degree of curvature of the index finger as shown in Figure 2B. The curvature of each finger is represented by the angle between two vectors. Specifically, the degree of curvature of the middle finger is represented by the angle between vector 0_9 and vector 10_12, the degree of curvature of the ring finger is represented by the angle between vector 0_13 and vector 14_16, and the degree of curvature of the little finger is represented by the angle between vector 0_17 and vector 18_20. The motion of the thumb and the average of the remaining four fingers are treated as separate sub-motion, and the following evaluator takes the maximum averaged angle and the averaged velocity as eigenvalues (F21 and F22).

In item 26–30, DFPS is used to measure hand grip force, and a certain threshold is set for effective gripping, and the threshold in this study is set as the minimum force to be detected by the DFPS. Due to the different handles, the positions of the force on the DFPS are different. Hook grasp., cylinder grasp and Sphere grasp exert force on the whole palm, while lateral pinch mainly exerts force on thumb and index finger, and Pincer grasp exerts force on thumb, index finger and middle finger. Therefore, it is not feasible to calculate the grip strength only by the sum of the thumb and index finger. This study proposes to calculate the total grip force of the hand (affected side/healthy side) by calculating the sum of the forces of each sensing unit in the inductive area (F23–F27).

Coordination and velocity [item (31)–(33)] (Huo et al., 2020): based on the measured fingertip data, the velocity and acceleration of the fingertip can be obtained, and average velocity and average acceleration are analyzed as feature (F28) for tremor classification. The characteristics of finger-nose dysmetria (F29) are mainly manifested in two aspects, namely, the ratio of the horizontal distance from the fingertip position to the left and right shoulder joints β, and the fingertip-to-nose movement length ratio γ as shown in Eq. 4.


(4) $$\gamma =\frac{\Delta d}{d_{FON}}$$

where Δd represents the distance from the real-time position of the fingertip to the nose in the vertical direction, $d_{FON}$ is the distance from the original position of the fingertip to the nose in the vertical direction. The hands are naturally placed at the sides of the body as the original position. The greater the movement length ratio, the better the patient’s ability to control the affected limb. The characteristic of finger-nose speed (F30) is mainly expressed by the maximum value of the movement time and the speed of the hand movement.

### 2.4 Assessment method

There is no obvious standard boundary for the classification of patients’ motor function grades, and the clinician’s assessment method is fuzzy. The use of fuzzy mathematical methods for assessment and analysis appears to align more naturally with objective facts. Therefore, this study proposes a multi-group fuzzy inference system for rehabilitation assessment (FISRA) based on the experience of rehabilitation physicians. To standardize the assessment system, the movement information of the healthy limb is collected simultaneously with the affected limb. The ratio/deviation of the sum of the movement/distributed force on the affected side to that on the healthy side is used as the eigenvalue to design the assessment method for the grades of the affected limb. It is important to note that joint movement and force application criteria differ for each subject.

The inputs to FISRA were the ratio of affected side ROM (AROM) to healthy side ROM (HROM), the ratio of affected side MV (AMV) to healthy side MV (HMV), and the SD. The trapezoidal membership function is adopted, as shown in Eq. 5. The output represents the assessment score using the triangular membership function, as shown in Eq. 6. As Elbow flexion in item (10), the FISRA inputs are AROM/HROM, AMV/HMV and SD, using trapezoidal membership function. and the output is rehabilitation assessment score, using triangular membership function. The assessment level is lower when the average speed AMV is slower, the AMV/HMV is smaller, and the SD is larger. Conversely, the assessment level is higher when the AROM/HROM is larger, the average speed AMV is higher, the AMV/HMV is larger, and the SD is smaller.


(5) $$f\left(x\right)=\{\begin{array}{l} 0\;\left(x\leq a\right)\\ \frac{x-a}{b-a}\;\left(a\leq x\leq b\right)\\ 1\;\left(b\leq x\leq c\right)\\ \frac{d-x}{d-c}\;\left(c\leq x\leq d\right)\\ 0\;\left(x\geq d\right) \end{array}$$

(6) $$f\left(x\right)=\{\begin{array}{l} 0\;\left(x\leq e\right)\\ \frac{x-e}{f-e}\;\left(e\leq x\leq f\right)\\ \frac{g-x}{g-f}\;\left(f\leq x\leq g\right)\\ 0\;\left(x\geq g\right) \end{array}$$

For item (12) Hand to lumbar spine, the extracted feature is the length ratio *α* of the hand to hip movement. The input is the length ratio of the affected side to the length ratio of the healthy side (E*α*/H*α*). A trapezoidal affiliation function is used. The greater the E*α*/H*α*, the greater the distance from the hand to the hip on the affected side, and the lower the assessment score.

For the items containing multiple sub-motions, each sub-motion is fuzzy evaluated first, and a separate FISRA is established. Then the evaluation results of multiple sub-motions are combined to evaluate the motor function score of the affected upper limb, as shown in Eq. 7.


(7) $$Ra=\{\begin{array}{l} 0\;\exists \;FIS_{i}=0\\ 1\;\mathrm{else}\\ 2\;\forall \;FIS_{i}=2 \end{array}$$

- If the score of multiple sub-motion is 2, the final evaluation score is 2.
- If one of the multiple sub-motion scores is 0, the motion evaluation score for that action is 0.
- All other assessments were scored 1.

**Figure 3:**
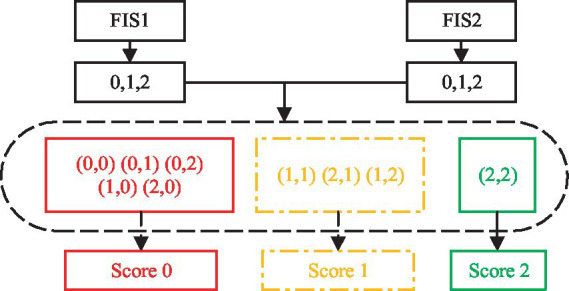
Two sets of FIS.

Item (13), (16), and (20) each have two actions. For example, (13) involves shoulder flexion 0–90° with the elbow fully extended including sub-motion M4 and M5. The features of the two sub-motions are extracted, FIS1 and FIS2 are used for evaluation, and the AROM/HROM, AMV/HMV and SD of each sub-motion are input into the model, the two evaluation scores are output respectively, and then the results of the two FIS are processed according to Eq. 7, as shown in Figure 3.

Items 14, 15, 17, 19, and 21–23 contain three sub-motions respectively, then three FISs are adopted. The AROM/HROM, AMV/HMV and SD of each sub-motion are input into a model respectively, the three evaluation scores are output, and then the results of the three FISs are processed according to Eq. 7.

Item (24) and (25) contains the motion of five fingers. Two FISs are used for the average results of the thumb and the other four fingers, respectively. The ratio of the maximum bending angle of the affected side to the maximum bending angle of the healthy side (AAngle/HAngle) and the AMV/HMV are input into each FIS, and then the evaluation scores are output. The larger the AAngle/HAngle, the larger the MV, the higher the evaluation score. Then the results of the two FIS are processed according to Eq. 7.

In item (26)–(30), the feature extracted by each item is the sum of the forces of the sensing unit. The input of the FIS is the sum of the distributed forces on the affected side to the healthy side (AFsum/HFsum). The greater the AFsum/HFsum, the higher the level of hand motor function. The lower the AFsum/HFsum, and the lower the level of hand motor function.

In the process of fingertip pointing to the nose in action 31, the ratio of the movement characteristics of the fingertip on the affected side to those on the unaffected side is calculated, AMV/HMV and AMA/HMA are input into each FIS to obtain the final assessment score. For item 32 finger-nose dysmetria, the characteristics of the affected side/unaffected side are calculated. The vertical distance A*β* of the affected side is divided by the vertical distance H*β* of the unaffected side. The movement length ratio of the affected side A*γ* is divided by the movement length ratio of the unaffected side H*γ*. These values are input into the FIS to obtain the assessment score. For item 33 speed, the ratio of AVmax (maximum velocity) on the affected side to HVmax on the healthy side is calculated and inputted into FIS. Then, the evaluation grade is obtained.

FIS adopts Mamdani fuzzy inference method, which is obtained by Cartesian product of fuzzy set. The defuzzification method is “centroid” to transform the fuzzy conclusion into a specific and accurate output process.

### 2.5 Human-computer interaction

**Figure 4:**
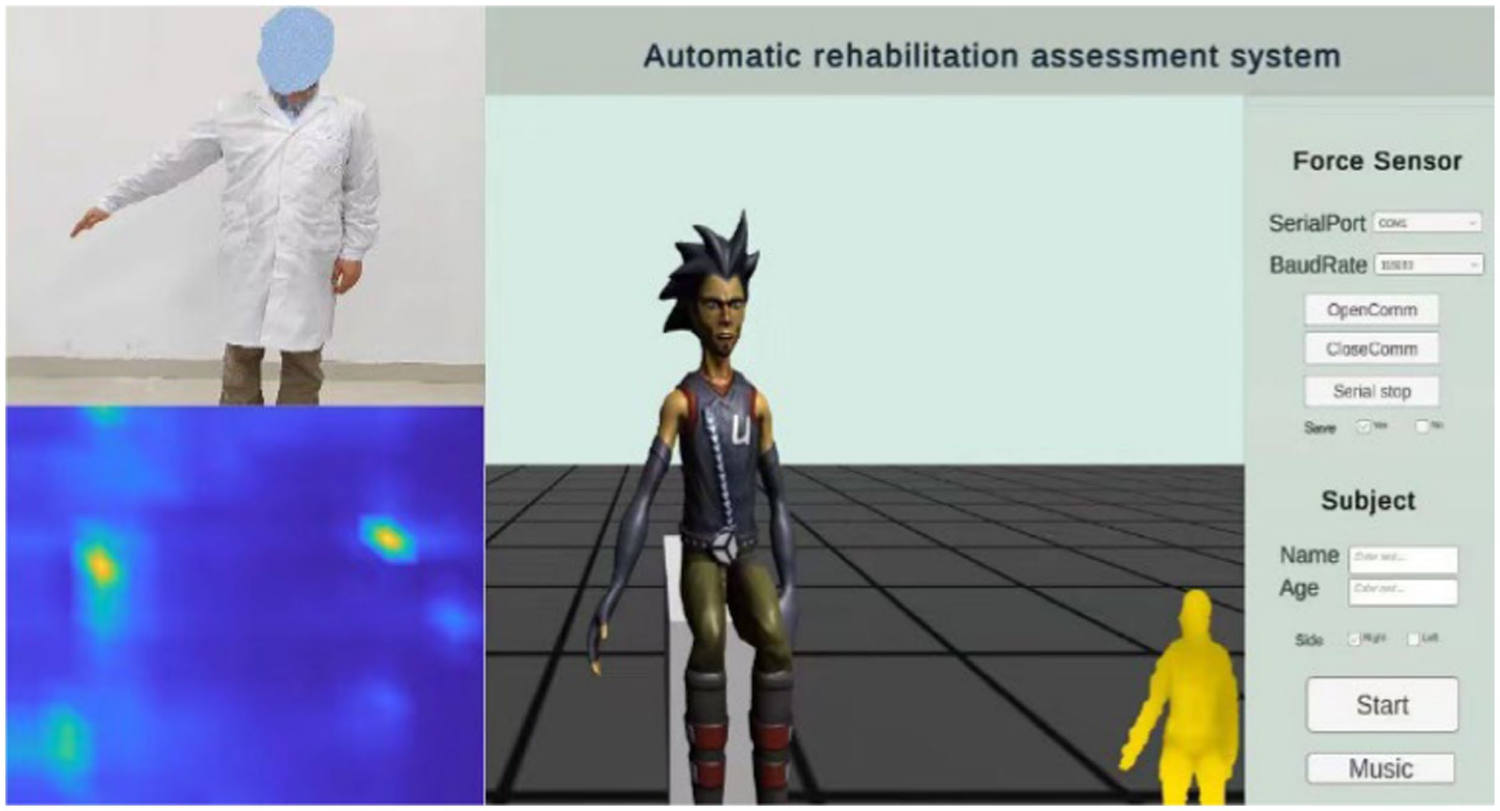
Human machine interface.

Interactive virtual environment can improve the enthusiasm and attention of patients. Based on previous research and the doctor’s recommendations, this study designed a human machine interface for assessing upper limb motor function. It includes motion-teaching videos, virtual feedback scenarios, and the distributed force sensor results, as shown in Figure 4.

The rehabilitation assessment teaching video is recorded by experienced rehabilitation physicians, including 20 actions (M1–M21) proposed in 2.1 Automatic FMA-UE. A part of the remaining time is set aside after each action, to ensure that the patient has enough time to complete the action. The patient’s appearance was neglected during illness. This study is based on the concept of paying attention to the patient’s mental health. The feedback video does not show the patient’s real image to protect the patient’s privacy. Instead, it uses an avatar to provide the patient with mirror feedback, display the subject’s movement posture, and show the subject’s real posture through a small window. In the virtual scene, arrows are added to indicate the direction of movement, and the voice is added to prompt the action content. Appropriate encouragement is given according to the patient’s completion (e.g., very good, come on, you are awesome, etc.) to further enhance the patient’s motivation to participate in the assessment of the movement for optimal exercise performance. At the same time, the scene is equipped with soothing background music to help relieve the patient’s mood.

The specific operating procedures are as follows: First, enter the patient’s name and age, select the affected side, and click the Start button. The video teaching starts, the patient imitates the movements demonstrated by the doctor, and Azure Kinect synchronously collects the patient’s postural information, mainly including postural measurements of items (3–17), (19–25) and (31–33). Music can be played simultaneously by clicking the Music button to create a favorable environment. Pause the video at the end of the pose acquisition. Then select the appropriate serial port, set the baud rate 115,200, and click to continue playing the video. Combine the video instruction to capture the force information of the items (26–30), click the save button to save the force information, switch the serial port using the pause button, and click the finish button when the acquisition is complete.

## 3 Experiments and results

### 3.1 Experiment setup

**Figure 5:**
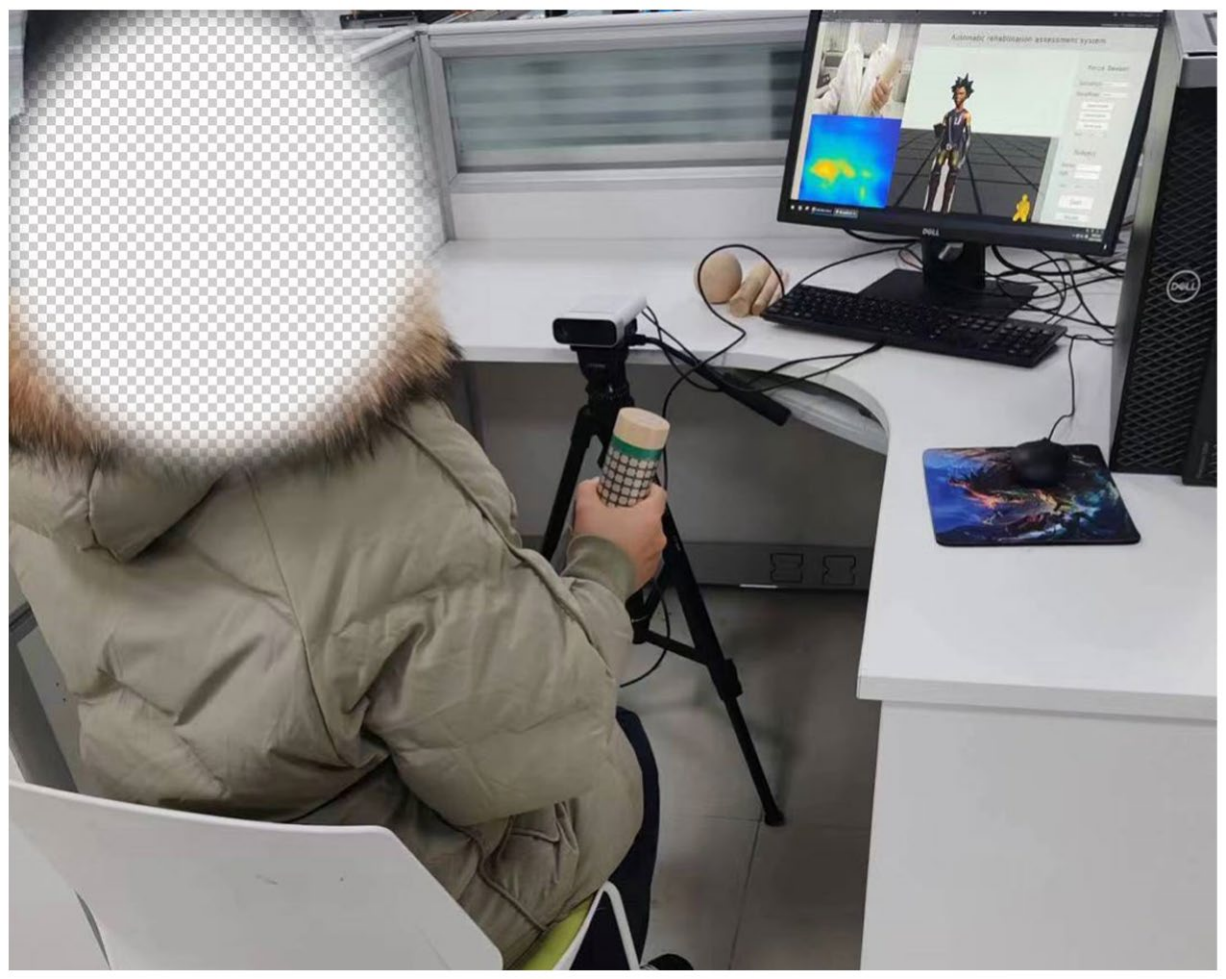
Experimental platforms.

The experimental equipment of the rehabilitation evaluation system mainly includes a computer, a monitor, an Azure Kinect, a tripod and a grasping tool attached with a distributed sensor [cylinder (diameter: *d* = 1 cm, *d* = 3 cm, *d* = 5 cm), ball, slice], as shown in Figure 5. The subjects sat on a chair without armrests, with the Azure Kinect placed 1.5 m directly in front of them, so that the subjects were in the center of the best visual field of the camera, and the grip tools attached with distributed sensors were placed on the table next to the subjects. According to the size of human palm, 16 rows and 16 columns DFPS is selected. Two hundred fifty-six sensing units are distributed in the square of 150 mm × 150 mm, and the size of each induction unit is 7.5 mm × 7.5 mm.

**Figure 6:**
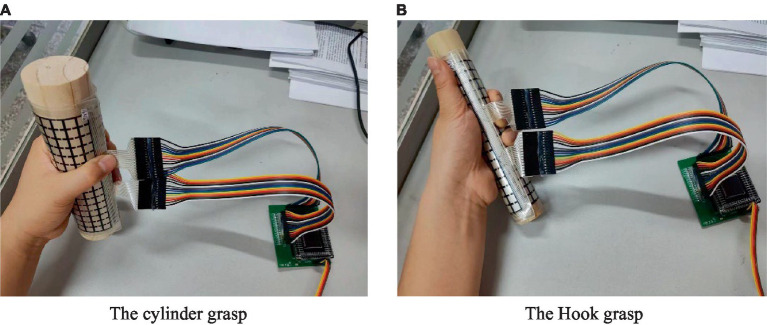
Measuring method of distributed force, **(A)** the cylinder grasp, **(B)** the hook grasp.

While the subject performed various movements according to Table 1, the Azure Kinect in combination with the MediaPipe measured the posture information of each joint of the upper limb and the hand on both the affected and the healthy side. The values of each feature used for the evaluation were then calculated. DFPS is attached to the gripping tool to measure the distributed force of the hand. During the hook grasp (26), the DFPS was attached to a cylinder with a 3 cm diameter. The subject’s hand was hooked to grasp the device with maximum force in the way of carrying a purse. The forces of both the healthy hand and the affected hand were measured. For the lateral pinch (27), DFPS was attached to the slice and placed between the thumb and index finger. The other four fingers were pinched together with the thumb to pinch the DFPS with the maximum force. The measured result was lateral pinch force. For the Pincer grasp (28), the DFPS was attached to the 1 cm-diameter cylinder. The measured hand grasps the device as if holding a pencil to measure the force. For the cylinder grasp (29), the DFPS is attached to a 5 cm diameter cylinder, and the hand grasps the device in the manner of holding a cup. For the Sphere grasp (30), the DFPS is attached to the spherical object and the device is held in the hand for force measurement. The cylindrical grasping and hook grasping posture are shown in Figure 6, panel (A) is the hand grasps the cylinder of the 5 cm in a cylindrical shape, panel (B) is the hand grasps 3 cm cylinder in the form of a hook.

**Figure 7:**
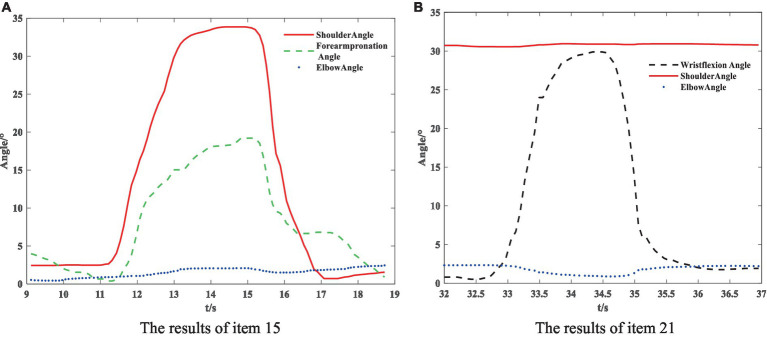
Posture measurement result, **(A)** the results of item 15, **(B)** the results of item 21.

### 3.2 Participant and protocol

Participants included 17 stroke patients (10 males, 7 females; Age: 58 ± 16.5 years) and 1 healthy subject, no severe cognitive impairment (MMSE score >15), and able to maintain a chair-sitting position. Patients with a Fugl–Meyer score below 10 were excluded due to the severity of their post-stroke condition, which hindered the collection of signals from the affected limb. Prior to data collection, all patients underwent evaluation by a rehabilitation technician and were tested for cognitive impairment. The patient’s statistical information is shown in the Table 2.

**Table 2:** The patient’s statistical information.

Subject	Gender	Affected side	Etiology	Time since stroke (month)	FME-UE
S1	M	R	Ischemic	1	35
S2	M	R	Hemorrhagic	3	29
S3	M	L	Ischemic	7	14
S4	M	L	Ischemic	1	16
S5	M	L	Hemorrhagic	2	27
S6	F	L	Hemorrhagic	13	53
S7	F	R	Hemorrhagic	4	25
S8	F	L	Ischemic	1	38
S9	F	R	Ischemic	5	42
S10	M	R	Ischemic	7	31
S11	M	R	Hemorrhagic	5	57
S12	M	L	Ischemic	2	20
S13	M	R	Hemorrhagic	1	41
S14	F	R	Hemorrhagic	3	47
S15	F	L	Ischemic	4	57
S16	F	L	Hemorrhagic	2	28
S17	F	L	Hemorrhagic	1	60

In this study, an experienced rehabilitation physician demonstrated and recorded each action of automatic Fugl–Meyer. They also provided a detailed explanation for each action. Before each data collection, the experimenter explained the experiment process to the patient. Then, they played the evaluation action video, and left enough time for the patient to practice the action. Finally, the experiment was conducted. Each subject was evaluated 6 times, each time at an interval of 10 min, to reduce the impact of the previous exercise on the next one. Both the affected and healthy limbs were tested with the same movements. It should be noted that the experiment will be stopped as soon as the subject reports any uncomfortable sensations. To reduce the influence of uncertain factors, four better results were selected for each patient. These results were used as the original data for the rehabilitation evaluation. A total of 72 groups of data were obtained.

### 3.3 Statistical analysis

To evaluate the accuracy of the proposed assessment method in this research, Bland–Altman analysis was applied to assess the agreement between the assessment results of this system (SFMA) and those of the rehabilitation physicians (RPFMA) Bland–Altman analysis calculates the limits of agreement between the two measurements, which are then visualized graphically.

Pearson correlation analyses were performed between the subjects’ SFMA and the total FMA-UE score (TFMA) to evaluate whether the SFMA score of the 30-item motor assessment method proposed in this research could replace the TFMA of the 33 upper extremity movements in the FMA-UE scale.

## 4 Results

### 4.1 Posture measurement results

**Figure 8:**
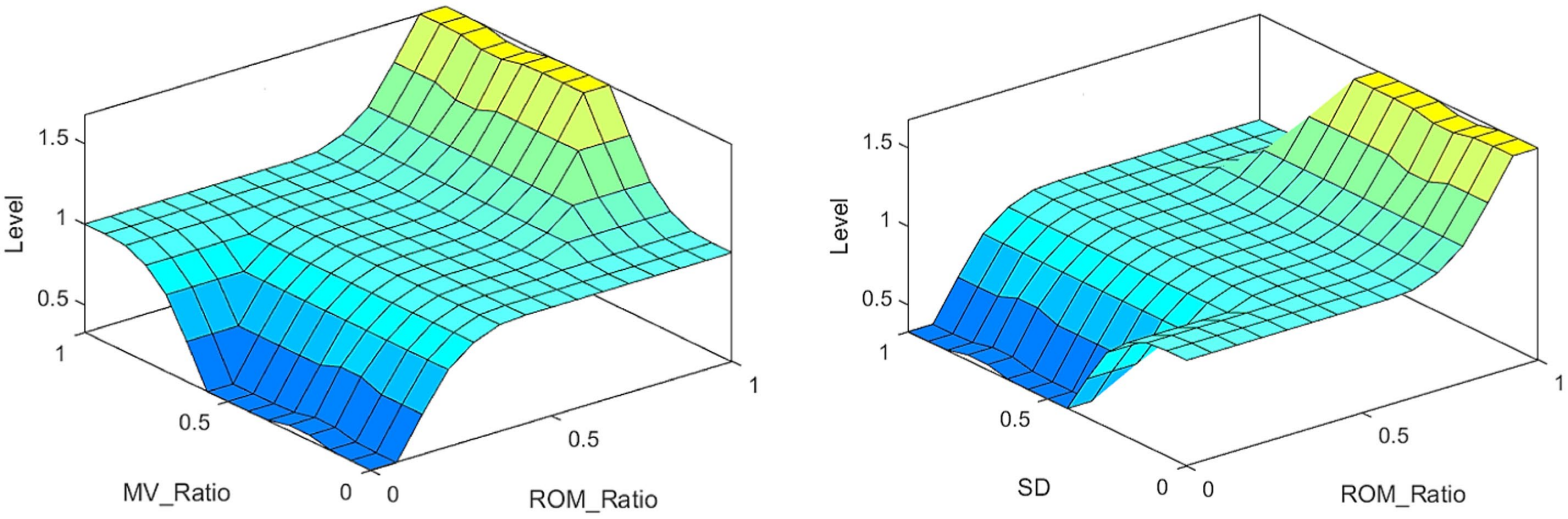
Fuzzy rules.

The partial results of the posture measurements are shown in Figure 7. Sub-figure (A) shows the results of the 15th item for a subject with a Fugl–Meyer score of 75. This item consists of a total of three movements. The graph displays the shoulder abduction angle (red solid line) with a maximum of approximately 34°, the elbow angle (blue dotted line) with a measured range of 0–2°, indicating that the elbow is in a state of straightening, and the angle for forearm rotation forward (green dotted line) with a range of 1–19°, indicating poor forward rotation ability. The results suggest that the subject is only able to partially complete the movement, and forward rotation of the forearm is essentially impossible. The fuzzy controller took AROM/HROM (ROM Ratio), AMV/HMV (MV Ratio), and SD as inputs and Rehabilitation Assessment Levels as output. Both inputs and outputs used three affiliation functions: low, medium, and high. The domains of ROM Ratio, MV Ratio, and SD were [0, 1], and the domain of the output was [0, 2]. The three inputs were represented as trapezoidal subordination functions with parameters [−0.4 −0.1 0.1 0.4], [0.1 0.4 0.6 0.9] and [0.6 0.9 1.1 1.4]. The output utilized three triangular membership functions for affiliation with parameters [0 0 1], [0 1 2] and [1 2 2]. The fuzzy rule is illustrated in Figure 8. The FISRA scored the shoulder abduction angle with 1 point, the elbow angle with 2 points, and the forward forearm rotation with 1 point. The final assessment result was 1 point, which was consistent with the doctor’s assessment.

Subfigure (B) displays the results of the 21st test maneuver for a subject with a Fugl–Meyer score of 82. The maneuver consisted of three total movements. The graph displays the wrist dorsiflexion angle (black dotted line) with a maximum of approximately 30°. The elbow angle (blue dotted line) is measured within the range of 0–3°, indicating a straightened elbow joint. The joint forward flexion angle (red solid line) is at an angle of about 31°, indicating better completion of the subject’s wrist dorsiflexion. The FISRA scored 2 for wrist dorsiflexion, elbow joint angle, and shoulder joint angle. The final assessment result for this movement was also 2, which was consistent with the doctor’s assessment.

### 4.2 Distributed pressure measurement results

**Figure 9:**
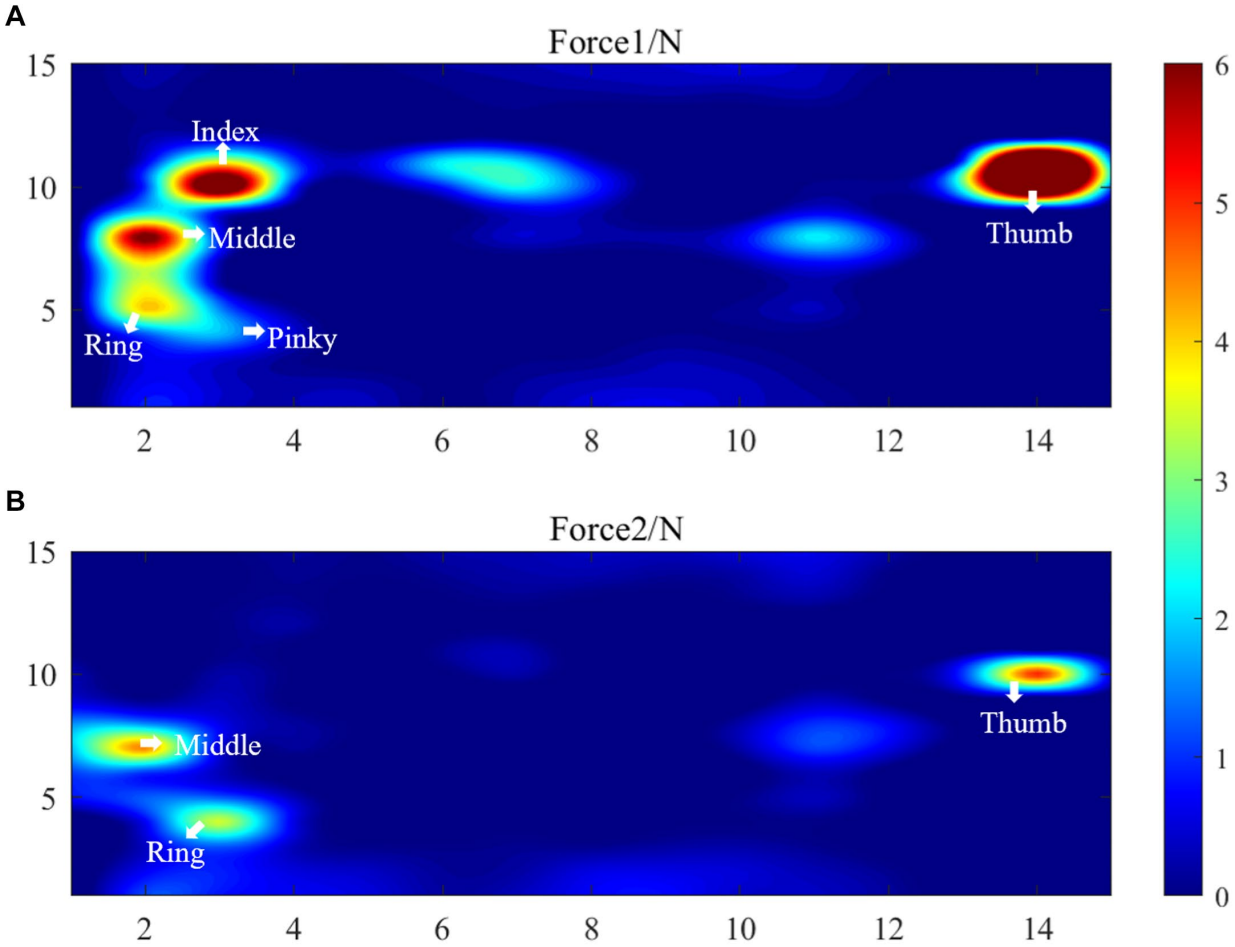
Distributed pressure measurement results, **(A)** Force1 (the maximum force information Force1 when grasping the cylinder with the healthy hand), **(B)** Force2 (the maximum force information Force2 when the affected hand grasps the 5 cm cylinder).

The results of the healthy hand were mirrored to facilitate comparison with the affected side. This put the fingers of the affected and healthy sides in the same approximate area. Figure 9 displays the distributed pressure measured by the cylindrical grasping device. Sub-figure (A) shows the maximum force information (Force1) when grasping the cylinder with the healthy hand. The force points of the thumb, index, middle, ring and little fingers are clearly visible, with maximum forces of 12.2 ± 1.3 N, 7.6 ± 1.2 N, 6.3 ± 1.6 N, 4.1 ± 2.0 N, and 3.6 N ± 2.6, respectively. Together with the force information for the rest of the hand, the total combined force of the hand is 68.2 ± 5.7 N.

Sub-figure (B) shows the maximum force information (Force2) when the affected hand grasps the 5 cm cylinder. This figure only shows the force information of the thumb, middle finger and ring finger. The maximum forces of these finger are 5.1 ± 2.7 N, 4.5 ± 1.5 N, 3.4 N ± 1.3 N, respectively. The total force of the palm sensing unit is 24.8 N. It shows that the patient’s index finger does not produce force information. Upon reviewing the patient’s medical records, it was found that the patient has motor dysfunction of the index finger joint on the affected side. The patient’s cylindrical grip motor function score on the affected side was assessed as 1 by FISRA, which was consistent with the physician’s evaluation.

### 4.3 Statistical analysis results

**Figure 10:**
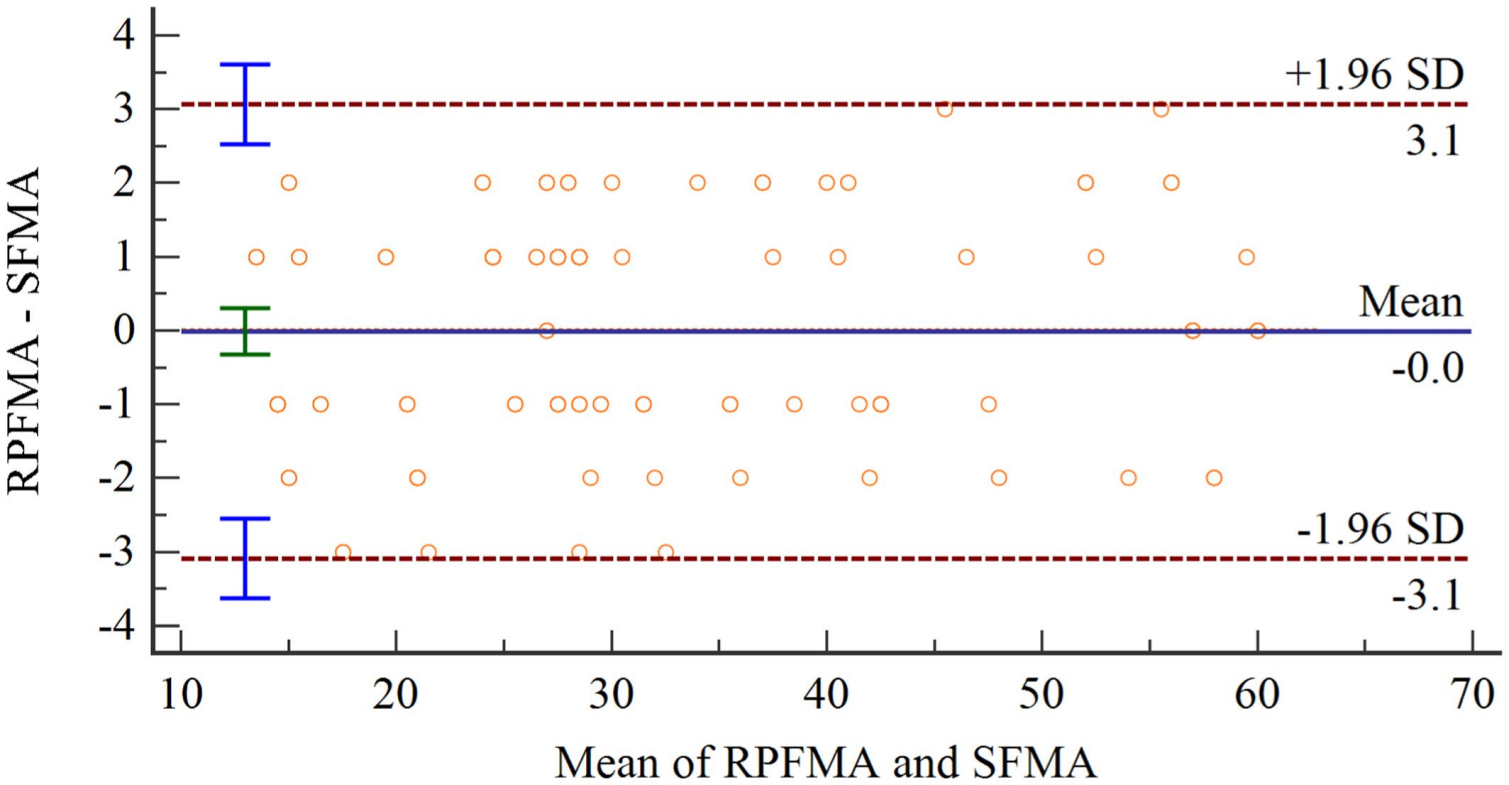
The Bland–Altman plot of the two assessment results.

The Shapiro–Wilk test was used to test the difference between SFMA and TFMA and ensure a normal distribution. Then Bland–Altman diagram was used to analyze the consistency of the two groups of data. As shown in Figure 10, the abscissa represents the average value of the two sets of data. The ordinate represents the difference between the two groups of data. The upper and lower brown horizontal dashed lines represent the upper and lower limits of 95% consistency. The middle blue solid line represents the average difference. The orange dotted line represents the average difference of 0. The arithmetic mean is −0.0102, 95% confidence interval (CI) is −0.3249 to 0.3054. As can be seen from the figure, there is no point outside the 95% CI, so the consistency between the two evaluation methods is good.

**Figure 11:**
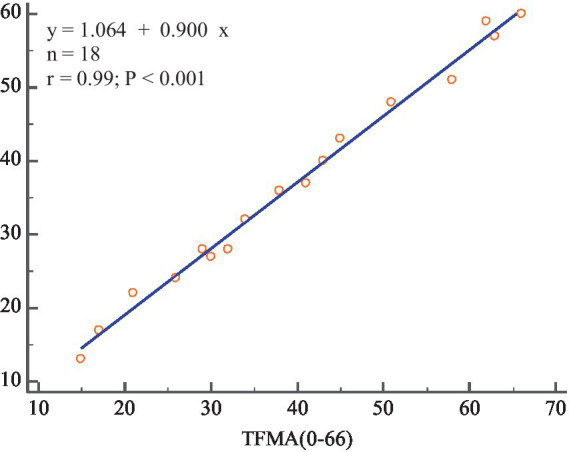
The correlation between SFMAM and TFMA.

The SFMA mean (SFMAM) of each subject was calculated, and a total of 18 data sets were obtained. The Pearson correlation analysis between SFMAM and TFMA of the subjects’ upper limbs was shown in Figure 11, *r* = 0.99 ~ *p* < 0.001. There was a very significant positive correlation between them.

## 5 Discussion

Rehabilitation assessment can evaluate the severity, development trend, and prognosis of patients with dysfunction. It provides an objective basis for formulating rehabilitation treatment plans. It also observes the development and changes of disability to evaluate the effect of rehabilitation treatment, and develops new and more effective means of rehabilitation treatment. Rehabilitation treatment often starts with a rehabilitation assessment and ends with another assessment. Therefore, the rehabilitation assessment of stroke patients is very important.

Rehabilitation physicians use protractors, grip dynamometers, and other equipment for manual measurement and evaluation. The measurement method is related to the doctor’s preference, and the measurement results depend on the doctor’s habits. The upper limb rehabilitation assessment time lasts more than 30 min. This longer time results in fewer clinical and scientific rehabilitation assessment methods. Most of the formalized rehabilitation assessments in hospitals are assessed only once at inpatient and once at discharge.

**Figure 12:**
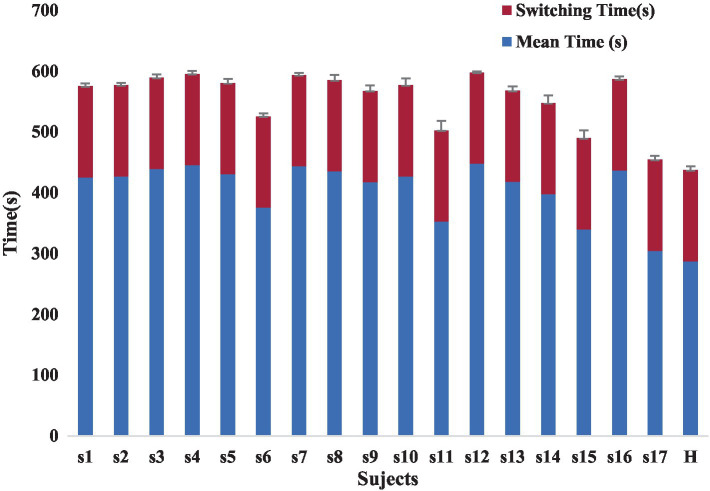
The rehabilitation assessment time.

The lack of uniform evaluation standards among rehabilitation healthcare systems, the lack of reasonable evaluation indexes for rehabilitation physicians, and the lack of a way to compare patient treatment outcomes limit the development of rehabilitation therapy technology. Our proposed automated Fugl-Meyer system, including Azure Kinect and distributed pressure sensors need not be worn. The device uses automated measurements where the patient only interacts with the display without human intervention, improving standardization and accuracy of measurements. Each assessment takes less than 10 min, greatly improving the efficiency of rehabilitation assessment. Patient rehabilitation assessment time included the time for the patient to perform the assessment actions and the device switching time. The device switching times were all 3 min, and the time consumed by the patients varied, as shown in the Figure 12. At the same time, the addition of virtual rehabilitation assessment scenarios can greatly improve the enthusiasm of patients to participate in rehabilitation assessment.

Various technologies can be used to acquire human motion information, including data gloves, sEMG, IMUs, robots, and Kinect. Data gloves can accurately capture hand opening and closing information. However, they may be difficult to wear for patients with muscle contractures.

sEMG can measure the EMG signals of a limited number of muscles. Since human movement is the result of the joint action of upper limb muscles, sEMG cannot fully characterize the overall movement of the subject. Additionally, sEMG is susceptible to interference and requires close proximity to the muscle being measured. Measuring the sEMG signals of the active muscles in the shoulder joint necessitates the removal of clothing, making the measurement inconvenient (Merletti et al., 2021). The inertial measurement unit used to measure movement must be worn in multiple locations and is also prone to displacement, causing discomfort to the subject (Li and Yu, 2023). Robots for rehabilitation assessment can be expensive and limited to hospitals or large communities. However, the Kinect system offers a portable, cost-effective, and practical alternative that does not require markers and is convenient for patients to use. The Azure Kinect is even more optimized and accurate than the previous versions. It is important to prioritize patient comfort and universality when selecting an assessment device. This paper selects Azure Kinect for upper extremity joint acquisition. However, it should be noted that Azure Kinect has limited hand joint acquisition capabilities and can only track four joint information points: wrists, hand tip, palm center, and thumb. To enhance the accuracy of the automatic evaluation system, Azure Kinect is combined with MediaPipe to acquire hand posture.

Lee’s device, which uses Kinect combined with FSR sensors for rehabilitation assessment, only measures the force exerted on the index finger and thumb fingertip. This limited measurement does not accurately reflect the force exertion in other parts of the hand. If a subject is unable to exert force on the index finger, the hand grip force information cannot be accurately measured.

To increase the generalizability of automated devices for rehabilitation assessment, this study proposes using a large-area distributed flexible pressure sensor to measure hand force during different gripping maneuvers. The flexible pressure sensor is distributed, thin, and easy to bend. It can be attached to various grasping tools to measure hand force information during different grasping modes, such as hook grasp., lateral pinch, pincer grasp., cylindrical grasp., and spherical grasp. The sensor is distributed and has a large area to test the force distribution of the entire palm. This allows for a more detailed and accurate measurement of the force exerted by the hand at each location. Figure 9 displays the force information of the entire palm on the healthy side. The five fingers’ tips exert a more pronounced force, and a healthy individual’s maximum fingertip force is approximately 12.2 N. Besides the fingertip’s tip, force information is also present at the root of the index finger, the greater and lesser pisiform areas. Therefore, the distributed sensor proposed in this study covers a larger area and requires less interference without the need for the user to wear it. Users can grasp objects naturally, without the need for a demanding grip position.

The assessment criteria for patients may vary due to differences in joint strength and range of motion. This study extracted characteristic information, such as range of motion, speed, length ratio, angle, acceleration, and distribution force. The ratios of features or deviations between the affected and healthy sides were entered into a single/multigroup fuzzy logic assessment model for rehabilitation evaluation. This increases the standardization of the rehabilitation assessment system and reduces the impact of individual differences on the results.

The study proposes an automatic rehabilitation assessment system for upper limb motor function based on posture and distributed force measurement. The system only requires Azure Kinect and an array of DFPS to be connected to a computer and used in conjunction with the relevant software. It is simple to operate, easy to install, portable, and inexpensive, making it suitable for home rehabilitation assessment. Because rehabilitation training is a long-term process, it is not practical or cost-effective to conduct it exclusively in the hospital. During the recovery and after-effects period of a stroke, it is important to not only receive training in the hospital but also to pay attention to rehabilitation training at home. This training lacks the guidance of a doctor, so it is crucial to focus on rehabilitation assessment. An automated rehabilitation assessment device is essential. The automated assessment system proposed in this paper can realize safe and efficient home rehabilitation training and assessment, combined with the virtual rehabilitation training scenario previously proposed by the authors (Bai and Song, 2019).

The combination of Azure Kinect and MediaPipe can improve the accuracy of hand posture tracking by largely reducing occlusions and singularities. However, it cannot completely eliminate them. Occlusions can still occur if the shoulder, elbow, wrist, and Azure Kinect are in a straight line. Some scholars have proposed using Leap Motion for hand tracking. However, its hand tracking area is limited. Additionally, rehabilitation assessment is a dynamic process, and the tracking accuracy of Leap Motion decreases under high dynamic conditions. Therefore, dynamic high-precision tracking of hand joints remains a challenging problem to solve.

## 6 Conclusion

This research proposed an automatic assessment system for the motor function of hemiplegic upper limbs. The system can automatically assess the motor function of 30 movements on the FMA scale by measuring posture and distribution force. By comparing the posture and distribution force information between the affected and healthy sides, the influence of individual differences on the assessment results is reduced. The experiment on automated assessment with 17 participants demonstrated a significant correlation (*r* = 0.99, *p* < 0.001) between the results of the automated assessment system and those of the physician’s assessment. This research lays the foundation for standardizing and unifying the automatic rehabilitation assessment system.

## Data availability statement

The raw data supporting the conclusions of this article will be made available by the authors, without undue reservation.

## Ethics statement

The studies involving humans were approved by Nanjing Tongren Hospital Science and Ethics Committee. The studies were conducted in accordance with the local legislation and institutional requirements. The participants provided their written informed consent to participate in this study. Written informed consent was obtained from the individual(s) for the publication of any potentially identifiable images or data included in this article.

## Author contributions

JB: Conceptualization, Data curation, Formal analysis, Funding acquisition, Investigation, Methodology, Project administration, Resources, Software, Supervision, Validation, Visualization, Writing – original draft, Writing – review & editing. GL: Data curation, Software, Writing – original draft. XL: Writing – review & editing. XW: Conceptualization, Supervision, Writing – review & editing.
